# Changes in Serum Biomarkers of Oxidative Stress in Cattle Vaccinated with Tick Recombinant Antigens: A Pilot Study

**DOI:** 10.3390/vaccines9010005

**Published:** 2020-12-24

**Authors:** Marinela Contreras, Camila Peres Rubio, José de la Fuente, Margarita Villar, Octavio Merino, Juan Mosqueda, José Joaquín Cerón

**Affiliations:** 1 Interdisciplinary Laboratory of Clinical Analysis (Interlab-UMU), Campus of International Excellence Mare Nostrum, University of Murcia, Campus de Espinardo s/n, 30100 Murcia, Spain; camila.peres@um.es (C.P.R.); jjceron@um.es (J.J.C.); 2 SaBio, Instituto de Investigación en Recursos Cinegéticos, IREC (CSIC-UCLM-JCCM), Ronda de Toledo 12, 13071 Ciudad Real, Spain; jose_delafuente@yahoo.com (J.d.l.F.); margaritam.villar@uclm.es (M.V.); 3 Center for Veterinary Health Sciences, Department of Veterinary Pathobiology, Oklahoma State University, Stillwater, OK 74078-2007, USA; 4 Biochemistry Section, Faculty of Science and Chemical Technologies, and Regional Centre for Biomedical Research [CRIB], University of Castilla-La Mancha, 13071 Ciudad Real, Spain; 5 Faculty of Veterinary Medicine, University of Tamaulipas, Tamaulipas 87000, Mexico; mero840125@hotmail.com; 6 Faculty of Veterinary Medicine, Autonomus University of Querétaro, Santiago de Querétaro 76010, Mexico; joel.mosqueda@uaq.mx

**Keywords:** cattle, tick, vaccine, antioxidant, biomarker, oxidant, antibody

## Abstract

Tick vaccination is an environmentally friendly alternative for tick control, pathogen infection, and transmission. Tick vaccine protection is sometimes incomplete, which may be due to problems in the stability, conformation, and activity of antibodies. This might be related to oxidative stress, but more studies are needed about the possible relationships between oxidative stress and immune function. The objective of this study was to evaluate and compare various serum biomarkers of antioxidant response and oxidative damage in cattle vaccinated with two recombinant antigens, the chimera of Subolesin- BM95 (homologue antigen of BM86)-MSP1a and BM86, and a control consisting in the adjuvant of the vaccines. Cupric reducing antioxidant capacity (CUPRAC), ferric reducing ability of the plasma (FRAP), trolox equivalent antioxidant capacity (TEAC), total thiol concentrations, and uric acid were evaluated in serum to determine the antioxidant response. To evaluate oxidative status, ferrous oxidation-xylenol orange (FOX), total oxidant status (TOS), advanced oxidation protein products (AOPP) and hydrogen peroxide (H_2_O_2_) concentrations in serum were determined. In addition, correlations between biomarkers of oxidative stress and antibody titers were evaluated. A significant decrease in all antioxidant biomarkers, with exception of thiol, and also a decrease in the oxidant markers TOS, AOPP and H_2_O_2_ was observed in cattle vaccinated with BM86, that also showed the highest antibody titers response whereas no significant differences in any of the biomarkers were detected in the Subolesin-Bm95-MSP1a and control groups. In addition, the dynamics of Cuprac and H_2_O_2_ with time showed significant differences between the groups. Although this is a pilot study and the results should be interpreted with caution and corroborated by studies involving a large number of animals, our results indicate that, in our experimental conditions, those vaccines able to induce a lower oxidative stress produce a higher concentration of antigen-specific antibodies. Overall, the results of the study provided information on the behavior of different biomarkers related to antioxidant defense, and the oxidative damage in cattle in response to vaccination.

## 1. Introduction

Ticks (Acari: Ixodida) are obligate hematophagous arthropod ectoparasites that are distributed worldwide and transmit pathogens causing diseases in humans and animals [1,2]. Among ectoparasite arthropod vectors, ticks are considered to be second worldwide to mosquitoes as vectors of human diseases, and the most important vector of diseases that affect the cattle industry [2,3]. Control methods are based on the use of acaricides and repellents with associated drawbacks such as selection of arthropod-resistant strains and contamination of both the environment and animal products [4,5]. 

Tick vaccines are an environmentally friendly alternative to acaricides for the control of tick infestations and the infection and transmission of tick-borne pathogens, and research on tick vaccine development is more advanced than that reported for other major ectoparasites. [6,7]. Therefore, tick vaccine research may provide models for the development of vaccines against other arthropods [8,9].

The protective mechanism of tick vaccines is based on ticks feeding on immunized hosts ingesting antibodies specific for the target antigen. This would lead to the formation of the antibody–antigen complex that should reduce the levels and biological activity of the target protein and interact with conserved epitopes in other proteins, resulting in reduced tick feeding, development, and reproduction [10,11,12].

Humoral immunity developed by vaccines play a role in the protective response against ticks and the pathogens they transmit [6,8], but the protection provided by vaccines sometimes is incomplete and the mechanisms involved are still poorly understood [13,14]. Some studies concluded that conformational stability of immunoglobulins type G (IgG) was determinant for a longer stability, viability and activity of antibodies affecting their protective capacity [15,16]. Furthermore, this conformational stability can be significantly limited by exposure to factors known to contribute to protein damage such as oxidative stress [17]. Oxidative stress occurs when there is a marked imbalance between the production of reactive oxygen species (ROS) and their removal by antioxidants [18]. Previous studies suggest that oxidants had a significant influence on interactions between antigens and antibodies, decreasing antibody activity, which can adversely affect immune function [19]. However, up to now there are no studies on the oxidative status in serum of immunized animals with tick recombinant proteins and more studies are needed to fully understand the possible relationships between oxidative stress and immune function.

The main aim of this study was to evaluate and compare the possible changes in biomarkers of oxidative stress, including antioxidants and oxidants, in serum of cattle after vaccination with recombinant tick antigens. 

As antioxidant biomarkers, three assays commonly used to evaluate the total antioxidant capacity (TAC) were included in this study: trolox equivalent antioxidant capacity (TEAC), cupric reducing antioxidant capacity (CUPRAC), and ferric reducing ability of plasma (FRAP), as well as two individual antioxidants, uric acid and thiol [20,21,22]. Regarding oxidant status, it was evaluated using assays for ferrous oxidation-xylenol orange (FOX) levels, total oxidant content (TOS), advanced oxidation protein products (AOPP) and hydrogen peroxide (H_2_O_2_) [23]. 

## 2. Materials and Methods 

### 2.1. Experimental Design

The test subjects were seven-month-old, crossbred calves (*Bos taurus* × *Bos indicus*), with feeding based on pasture buffel grass (*Pennisetum ciliare L*.) from the spring season ad libitum. The average body weight of the animals was 189.4 ± 3.92 kg, and they had no previous exposure to ticks, and no evidence of concurrent vector-borne or non-infectious systemic diseases. Animals were randomly assigned to 3 groups of six animals each, two groups were immunized with recombinant *Rhipicephalus microplus* antigens and a control group was immunized with an adjuvant–saline alone (placebo) on days 0 (T0) and 28 (T1). Blood samples were collected at T0 immunization and fifteen days after the last one (day 42 (T2)). Animals were considered healthy after physical examination during routine check-ups. This study was carried out in strict accordance with the Guide for Care and Use of Laboratory Animals for the University of Queretaro and the protocol was approved by the Committee on the Ethics of Animal Experiments (Permit no: 23FCN2012).

### 2.2. Production of Recombinant Subolesin Antigens and Vaccine Formulation

Plasmids containing the coding Subolesin-BM95 MSP1a fusion proteins (SUB-BM95) were transformed into *E. coli* BL21 Star™ (DE3) One Shot^®^ cells (Invitrogen-Life Technologies, Inc., Grand Island, NY, USA) to express and purify SUB-BM95 as described previously [24,25]. The SUB-BM95 chimeric protein is based on SUB from *R. microplus* Media Joya strain and conserved BM86/BM95 protective epitopes. The recombinant BM86 (from *R. microplus* Media Joya strain) was secreted in *Pichia pastoris* and purified, as reported previously [26]. Recombinant proteins were adjuvated in Montanide ISA 50 V2 (Seppic, Paris, France) in a stable water in oil (W/O) vaccine emulsion (50 parts of aqueous phase and 50 parts of oily phase in the volume) in batch-by-batch processes using a high-speed mixer Heidolph DIA× 900 (Heidolph Elektro, Kelheim, Germany) at 8000 rpm and the vaccine was filled manually under sterile conditions in pyrogen-free glass bottles of 20 mL (Wheaton, Millville, NJ, USA) at a concentration of 100 µg/2 mL dose. Calves were immunized with 2 doses (days 0 (T0) and 28 (T1)) containing 100 µg/dose [27] of purified recombinant proteins formulated as described above. Negative controls were injected with the adjuvant–saline alone (placebo). Cattle were injected intramuscularly with 2 mL/dose using a 5 mL pyrogen-free plastic syringe and an 18G needle.

### 2.3. Determination of Serum Antibody Levels by ELISA

Before the first immunization (T0) and at the end of the experiment (day 42 (T2)), blood samples were collected from each calf into sterile tubes and maintained at 4 °C until arrival at the laboratory. Serum was then separated after centrifugation and stored at −20 °C until analysis, after that the samples were stored at −80 °C. Serum antibody titers were determined using an indirect ELISA [28]. Then 0.1 µg of each purified recombinant antigen (diluted in 50 µL of carbonate-bicarbonate buffer; Sigma–Aldrich, St. Louis, MO, USA) per well was used for overnight coating of high absorption capacity polystyrene microtiter ELISA plates at 4 °C. Plates were blocked with 200 µL/well of blocking solution (10% fetal bovine serum in phosphate-buffered saline (PBS), 137 mM NaCl, 2.7 mM KCl, 10 mM Na_2_HPO_4_, 1.8 mM KH2PO4 and pH 7.4) (Sigma–Aldrich). The sera were serially diluted to optimal dilution 1:1000 *v/v* in blocking solution. Plates were then incubated with 100 µL/well of diluted sera overnight at 4 °C, followed by three washes using PBS and 0.1% Tween 20 (PBST) and an incubation with PBS-diluted (1:10,000) rabbit anti-bovine IgG-HRP conjugates (Sigma–Aldrich) for 1 h at room temperature (RT). After three washes with PBST, the chromogenic reaction was developed with 3,3′5,5′-tetramethylbenzidine (Sigma–Aldrich), stopped with 50 µL/well of 3N H_2_SO_4_. Antibody titers were expressed as the O.D.450 nm values and compared between vaccinated and control groups, and between vaccination times, using a one-way ANOVA test (https://www.socscistatistics.com/tests/anova/default2.aspx) (*p* = 0.05; *n* = 6 biological replicates).

### 2.4. Antioxidant Biomarkers

Serum samples were maintained at –80 °C for 1 month, until analysis, to preserve the stability of the biomarkers [29]. Three assays previously validated for measuring TAC in serum of dogs were used: TEAC, CUPRAC, and FRAP [30]. TEAC was measured using horseradish peroxidase (HRP) to generate the radical 2,2′-azino-bis (3-ethylbenzthiazoline-6-sulfonic acid) (ABTS^•+^) (TEAC) [28]. CUPRAC and FRAP were measured by previously described assays [31,32]. Total thiol was measured according to the method described by Jocelyn (1987) and da Costa et al. (2006) [33,34]. Uric acid was measured as described Barranco (2019) [35]. All assays were performed using the Olympus AU400 (Olympus AU400 Automatic Chemistry Analyzer, Olympus Europe GmbH, Bellport, NY, USA).

### 2.5. Oxidative Biomarkers

TOS was measured by the method described by Erel (2005) [36]. FOX assay was measured by the method of Arab and Steghens (2004) [37]. AOPP were measured as described by Witko–Sarsat et al. (1996) [38]. Hydrogen peroxide (H_2_O_2_) was measured by the method described by Tatzber et al. 2003 [39]. Those assays were performed using the Olympus AU400.

### 2.6. Statistical Analysis

Results of the biochemical parameters were expressed as medians with interquartile range (IQR) and calculated using routine descriptive statistical procedures and software (GraphPad Prism 8.0.2 Software, San Diego, CA, USA). A two-way repeated measure ANOVA, incorporating sampling time (time) and group as experimental variables, followed by Sidak and Tukey’s post hoc test, was used to determine differences in analytes between the different sampling time-points and between the different immunizations, respectively. Correlations between the magnitude of increase in the biomarkers of oxidative stress and the antibody after administration of BM86 were determined using Spearman correlation analysis. The significance level used in each case was *p* < 0.05.

## 3. Results

### 3.1. Characterization of the Antibody Response in Vaccinated Cattle

**Figure 1:**
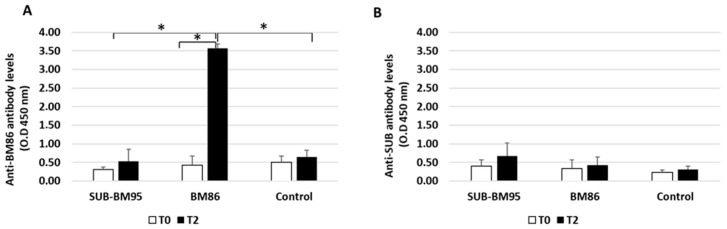
Antibody titers in vaccinated and control cattle against the recombinant BM86 (**A**) and Subolesin (**B**) proteins used for vaccination alone (BM86) or in combination (SUB-BM95). Serum samples were collected before 1st immunization (T0) and at the end of the experiment, two weeks after the last immunization (T2). Antibody titers in vaccinated cattle were expressed as the average ± S.D. OD450nm and compared between vaccinated and control groups, and between times in each group by ANOVA test (* *p* < 0.05; *n* = 6 replicates per treatment).

Anti-BM86 antibody titers increased significantly in cattle vaccinated with BM86, but not in cattle vaccinated with SUB-BM95 or controls after two immunizations (T2) (*p* < 0.05; Figure 1a). Antibody titers against BM86/BM95 did not increase in cattle vaccinated with SUB-BM95 when compared to controls (Figure 1A). In addition, no significant differences in anti-SUB antibody titers were observed in any of the groups when different immunization times and groups were compared (Figure 1B). These results confirmed the immunogenicity of BM86 and suggested that the combined SUB-BM86 chimeric antigen is not immunogenic, at least in cattle.

### 3.2. Changes in Antioxidant Biomarkers

**Figure 2:**
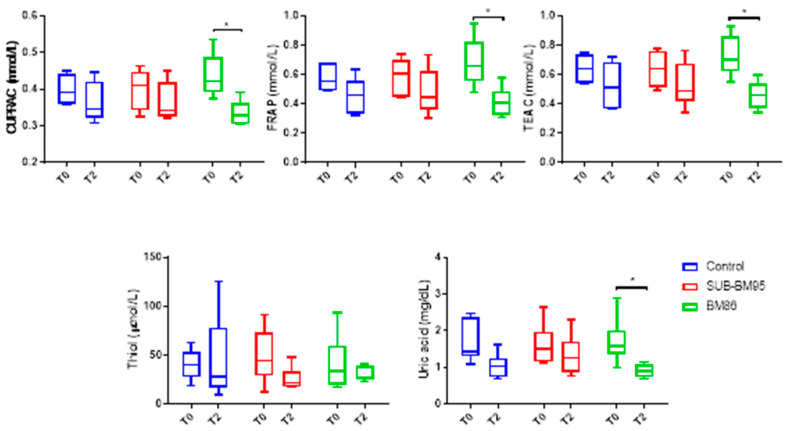
Values of antioxidant markers in vaccinated cattle with BM86, SUB-BM95, and control at T0 and T2. CUPRAC, cupric reducing antioxidant capacity; TEAC, trolox equivalent antioxidant capacity using horseradish peroxidase; FRAP, ferric reducing ability of plasma; total serum thiols and uric acid. The plots show median (line within box), 25th and 75th percentiles (box) and minimum and maximum values (whiskers). Asterisks indicate significant differences between times and immunization * *p* < 0.05.

The results of antioxidants at T0 and T2 are shown in Figure 2.

A significant effect of time was observed in CUPRAC (*p* = 0.0104) with a significant decrease in vaccination with BM86 at T2 (*p* = 0.0245). There was no significant effect of group (*p* = 0.95) and there was an interaction between time and group (*p* = 0.048).

A significant effect of time was observed in FRAP (*p* = 0.0061) with a significant decrease in vaccination with BM86 at T2 (*p* = 0.0250). There was no effect of o group (*p* = 0.6995) and there was no significant interaction between time and group (*p* = 0.066).

A significant effect of time was observed in TEAC (*p* = 0.0069) with a decrease in vaccination with BM86 at T2 (*p* = 0.0296). There was no group effect (*p* = 0.9573) and no interaction between time and group (*p* = 0.088).

Regarding thiol, there was no effect of time (*p* = 0.2787), group (*p* = 0.8602) neither significant interaction (*p* = 0.3120) between time and group.

A significant effect of time was observed in uric acid (*p* = 0.0035), with a decrease in vaccination with BM86 at T2 (*p* = 0.0412). There was no group effect (*p* = 0.7128) nor interaction between time and group.

### 3.3. Changes in Oxidant Biomarkers

**Figure 3:**
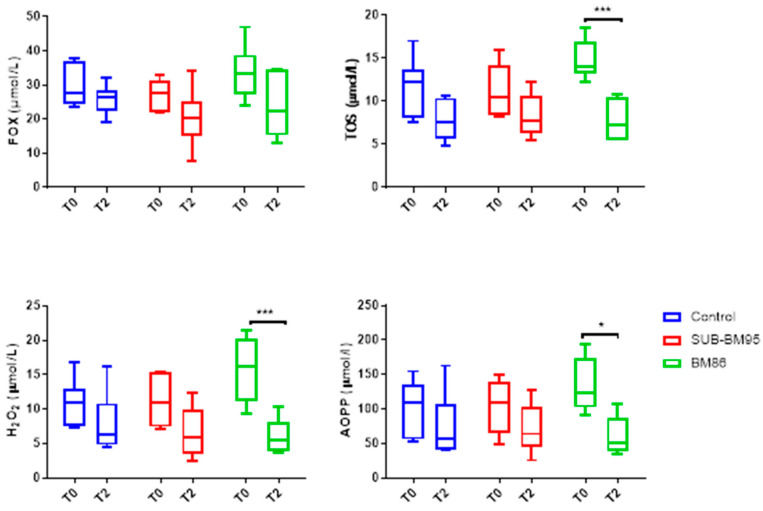
Values of oxidant markers in vaccinated cattle with BM86, SUB-BM95, and control at T0 and T2. FOX, ferrous oxidation-xylenol orange; TOS, total oxidant status; H_2_O_2_ hydrogen peroxide; AOPP, advanced oxidation protein products. Asterisks indicate significant differences between times and immunization * *p* < 0.05, *** *p* < 0.001.

The FOX, TOS, AOPP and H_2_O_2_ results are presented in Figure 3. FOX values had an effect of time (*p* = 0.0112), although no difference was observed between time-points in all groups (*p* > 0.05). In addition, there were no main effect of treatment (group) (*p* = 0.2268) and nor interaction between time and group (*p* = 0.5987).

TOS, H_2_O_2_ and AOPP were significantly affected by time (*p* = 0.0002, *p* = 0.0027, and *p* = 0.0083, respectively), with decreases in vaccination with BM86 at T2 (*p* = 0.0023, *p* = 0.0027, and *p* = 0.0336, respectively). These analytes showed no interaction between groups (*p* = 0.2772, *p* = 0.3810, and *p* = 0.7326, respectively). H_2_O_2_ showed an interaction between day and group (*p* = 0.037).

### 3.4. Correlation Study

The Spearman correlation coefficients and significance between the magnitude of changes of all biomarkers studied in the serum of vaccinated cattle and the antibody titers at T2 of BM86 were determined (Table 1). When the different oxidative biomarkers were evaluated, Spearman correlation coefficient revealed that antibody titers did not correlate with the magnitude of change in any of the biomarkers studied (all *p* ≥ 0.05).

## 4. Discussion

To the best of authors’ knowledge, no studies about changes in biomarkers of oxidative status in serum of cattle vaccinated with tick recombinant proteins have been previously published.

SUB and BM86 recombinant antigens from *R.microplus* were used because *R. microplus* is endemic of cattle in tropical and subtropical regions of the world, causing major economic losses to cattle producers through direct physical effects on the parasitized animals and indirectly through transmission of infectious disease [40]. Additionally, they were described and tested in previous vaccination trials, SUB is a highly conserved genetically and functionally across all tick species [41] and has shown protection for the control of multiple tick species infestations and pathogen infection and transmission [8] and BM86 was successful in the control of tick populations of *R. microplus* reducing the average fertility of adult ticks [24,25,26,27,28].

In previous studies, the SUB-MSP1a and BM95-MSP1a chimeras separately showed immunogenicity in the ELISA test [24]. In our experimental conditions, the antibody response corroborated the immunogenicity of the BM86 antigen [6,7], but not of SUB-BM95 since it did not produce significant increases in anti-SUB or anti-BM86 antibody titers. These results suggested an immune interference or blocking of immune reactive epitopes in the chimeric antigen [42] due to protein–protein interactions that have been reported for both BM and SUB proteins [41]. The antibody response to BM86 obtained in our study was in line with previous reports made in different herds of cattle showing a homogeneous peak in the antibody response, 2 weeks after the last immunization [8,43]. In addition, total IgG response with these antigens was not tested, but previous studies have shown higher total IgG levels associated with the adjuvant used in our study [44]. The pattern of antibody response of our experimental procedure allows us to study and compare the behavior of different biomarkers of the antioxidant defense and the oxidative damage in cattle, in response to vaccination with antigens with different immunogenicity.

In our study, the presence of an antibody response in animals vaccinated with BM86 was associated with a reduction in antioxidant biomarkers. This decrease in antioxidants may indicate that they are consumed as part of a defense mechanism against oxidative damage [45], which could contribute to IgG protection [46].

In the oxidant biomarkers, a decrease in TOS, AOPP and H_2_O_2_ was observed in those animals that produced antibodies in response to immunization being in the case of H_2_O_2_ the differences significant between groups with a higher decrease in the animals of the BM86 group. This result could be related to a decrease in protein oxidation and therefore an improvement in the antibody production. High level of oxidants would lead to oxidation and damage of the immunoglobulins [17,47], thus resulting in significant inhibition of antibody activity by altering the secondary or tertiary structure of antibodies [19]. The lack of changes in FOX could indicate that there is no product of lipid oxidation liberated during the immunization process.

In a previous study made in birds with parasites, a stronger immune response with higher immunoglobulin concentrations were related with low total antioxidant capacity (TAC) [48]. However, we did not find information in mammals about markers of oxidative status and immunoglobulin concentrations. Based on several previous studies [10,49] it could indicate that the protective mechanisms elicited by these vaccines are mediated by IgG antibody response. However, to the author`s knowledge, there were no studies about the influence of cell mediated immune responses induced by vaccines in changes in oxidative stress, and these should be evaluated in future trials.

There was no correlation between the magnitude of increase in the different biomarkers of oxidative status and the antibody concentrations in the BM86. Although this result should be taken with caution due to the low number of animals of the study, this could indicate that biomarkers of oxidative status and production of antibodies show different kinetics or dynamics during the immune response.

Based on the results of this study, the findings of lower values of oxidant biomarkers and, at the same time increases in specific IgG antibodies with the BM86, would indicate that the low values of oxidative agents would be related to less damage and oxidation of immunoglobulins, and possibly a more efficient production of those IgGs. o corroborate this hypothesis future experiments with a larger number of animals should be conducted in order to confirm two requirements: (1) if animals with high antibody titres usually have low values of oxidant compounds and also (2) to provide evidence that oxidant compounds compromises antibody function or half-life. Although there are previous studies that described both requirements [17,47,48] ideally this should be demonstrated also with the BM95 and BM86 antigens. If this could be demonstrated, vaccine formulations would benefit from producing an antioxidant response able to keep oxidant agents at low concentrations, in order to avoid oxidation of IgG.

In the present study, Montanide was used as adjuvant for Water-in-Oil (W/O) formulations, which is known to induce cellular and humoral antibody responses [50]. In our study, this adjuvant did not produce significant changes in the biomarkers of oxidative status evaluated. These results support that BM86 produced oxidative changes, which are not dependent on the adjuvant. Future experiments should address the limitations of our study regarding the use of negative controls only injected with saline, as well as the use positive controls, injected with agents other than vaccines that are known to induce oxidative stress.

## 5. Conclusions

The results of the study provided information on the behavior of different biomarkers related to antioxidant defense and the oxidative damage in cattle in response to vaccination. The antigen-specific IgG antibody response to vaccination with BM86 was higher than in SUB-BM95 and control groups, having the animals vaccinated with BM86 a significant decrease in antioxidant and oxidant biomarkers.

Based on these results, it could be concluded that a lower oxidative stress could be involved in the production of a higher level of antigen-specific antibodies. However, we are still in early stages of understanding how oxidative stress is related to vaccines. Therefore, this investigation should be considered as a pilot study and further studies are needed to investigate the trade-off between immune response and oxidative stress and how the vaccine formulation could preserve or modify this balance. Further trials with other vaccines and ideally using saline solution as a negative control and compounds known to produce oxidative damage as positive controls should be conducted in order to confirm these findings.

## Figures and Tables

**Table 1:** Spearman correlation coefficients and significance between the magnitude of change of the different analytes before and after vaccination and antibody titers studied in serum cattle vaccinated with BM86.

Heading	CUPRAC	FRAP	TEAC	Thiol	Uric Acid	FOX	TOS	H_2_O_2_	AOPP
Spearman r	−0.257	−0.222	−0.215	0.087	−0.198	0.202	−0.418	−0.214	−0.209
*p* value	0.303	0.376	0.390	0.729	0.430	0.421	0.084	0.393	0.404

CUPRAC, cupric reducing antioxidant capacity; FRAP, ferric reducing ability of plasma; TEAC, trolox equivalent antioxidant capacity; FOX, ferrous oxidation-xylenol orange; TOS, total oxidant status; H_2_O_2_, hydrogen peroxide, AOPP, advanced oxidation protein products.
